# The Allelopathic Inhibition of Submerged Macrophytes (*Ceratophyllum demersum* and *Myriophyllum spicatum*) in Response to Toxic and Non-Toxic *Microcystis aeruginosa*

**DOI:** 10.3390/microorganisms13122797

**Published:** 2025-12-08

**Authors:** Yuanyuan Tang, Shuwen Zhang, Jing Dong, Yuanpu Sha, Guiyu Chen, Xuejun Li, Xiaofei Gao, Huatao Yuan, Jingxiao Zhang, Penghui Zhu, Yunni Gao

**Affiliations:** 1 College of Fisheries, Henan Normal University, Jianshe Road, Xinxiang 453007, China; 2 Observation and Research Station on Water Ecosystem in Danjiangkou Reservoir of Henan Province, Nanyang 474450, China; 3 The National Ecological Quality Comprehensive Monitoring Station (Hebi Station), Hebi 458000, China; 4 Key Laboratory of Yellow River and Huai River Water Environment and Pollution Control, Ministry of Education, Xinxiang 453007, China

**Keywords:** submerged macrophytes, toxic and non-toxic *Microcystis aeruginosa*, ecological remediation, allelopathy, nutrients competition

## Abstract

The present study systematically explored the purification effects and response of submerged plants, *Ceratophyllum demersum* and *Myriophyllum spicatum*, on toxic and non-toxic strains of *Microcystis aeruginosa* via indoor co-culture experiments. The results showed that: (1) Both plants significantly inhibited the growth of *Microcystis* and reduced the concentration of chlorophyll-a (Chla) in the water by rapidly absorbing nutrients such as nitrogen and phosphorus, with no significant differences in the inhibition between toxic and non-toxic strains, indicating that nutrient competition might be the dominant mechanism for algal suppression. (2) *C. demersum* had higher nitrogen and phosphorus removal efficiency than *M. spicatum*, but the microcystins (MCs) released by toxic *M. aeruginosa* inhibited the nutrient removal capacity of both plants. (3) The plants promoted cell lysis of toxic *M. aeruginosa* and reduced extracellular MCs in the water while accumulating MCs internally, with *C. demersum* showing stronger MC accumulation and removal ability. (4) *Microcystis* stress activated the plants’ antioxidant defense systems, increased activities of SOD (Superoxide Dismutase) and CAT (Catalase), and caused membrane lipid peroxidation, increased content of MDA (Malondialdehyde), with toxic *M. aeruginosa* inducing stronger oxidative stress, and *M. spicatum* being more severely affected. (5) Plant species and algal toxicity jointly drove changes in the attached microbial community structure. The rhizosphere of *M. spicatum* specifically enriched Bdellovibrionota, suggesting a potential microbial predation pathway for algal suppression, while *C. demersum* was more associated with *Bacillus* and other microbes with allelopathic potential. In summary, *C. demersum* performed better in nutrient removal, toxin accumulation, and physiological tolerance. This study provides further theoretical support for using submerged plants to regulate cyanobacterial blooms and remediate eutrophic water bodies.

## 1. Introduction

With the rapid development of industry and agriculture, and the accelerated process of urbanization, a large amount of wastewater containing nutrients such as nitrogen and phosphorus is discharged into rivers and lakes, leading to the increasingly severe issue of water eutrophication globally. According to the “2023 China Ecological and Environmental Status Bulletin, among the 210 key monitored lakes (reservoirs) in China, 87.8% are still eutrophic (including 23.4% were mildly eutrophic, and 64.4% were moderately eutrophic), indicating that the nitrogen and phosphorus pollution load remains a significant challenge. Frequent outbreaks of cyanobacterial blooms are the most prominent feature of eutrophic waters. Among them, *Microcystis aeruginosa* is one of the most common and harmful dominant cyanobacterial species in freshwater bodies [1]. A large variety of *M. aeruginosa* have been reported that could produce secondary metabolites with strong hepatotoxicity—microcystins (MCs). MCs are a highly conserved class of cyclic heptapeptides with stable physicochemical properties, resistant to heat and acid, and are difficult to remove effectively with conventional drinking water treatment processes [2]. Among the more than 100 known MC variants, MC-LR (L-leucine-L-arginine structure) has attracted particular attention due to its highest frequency of occurrence and strongest cytotoxicity [3,4]. Currently, methods for controlling cyanobacterial blooms include physical and chemical approaches, such as mechanical harvesting, ultrasound, and algaecide application. However, these methods are often costly, can cause secondary pollution, and ultrasonic treatment, while breaking algal cells can promote the release of intracellular microcystins (MCs), potentially increasing toxin concentrations in water in short time [5]. Chemical methods, such as using copper sulfate or herbicidal algaecides, although fast-acting, are non-selective and can indiscriminately harm other aquatic organisms, disrupting ecological balance. Additionally, the accumulation of heavy metals like copper ions in sediments can result in long-term secondary pollution [6]. More critically, whether using physical or chemical methods, large-scale cell rupture triggers the instantaneous release of intracellular toxins, raising health risks at drinking water sources [7]. Therefore, seeking an environmentally friendly and sustainable biocontrol and remediation technology has become a current research focus. Submerged plants, as ‘ecological engineers’ of healthy aquatic ecosystems, play a key role in inhibiting algal growth and purifying water quality [8,9,10] and have thus been widely utilized in the restoration of aquatic ecosystems [11]. They mainly function through three ways: (1) Resource competition: Competing with algae for nutrients (nitrogen, phosphorus) and light in water, thereby inhibiting algal growth [12,13,14]; (2) Allelopathy: Releasing specific allelochemicals (such as phenolic acids, fatty acids, pyrogallic acid, ellagic acid, etc.) to directly inhibit the growth of algal cells or damage their photosynthetic systems [15]; (3) Attached microorganisms effects: The large leaf and stem surfaces of submerged macrophytes provide attachment substrates for diverse microbial communities (including bacteria, archaea, and protozoa). The “macrophyte-attached microorganism system” forms a complex microecosystem that can systematically degrade organic pollutants, including MCs [16,17].

*C. demersum* and *M. spicatum* are two common submerged macrophytes with strong purification capabilities. Previous studies have shown that *M. spicatum* could release ellagic acid and pyrogallic acid, thereby damaging the cell structure of *Microcystis*, inhibiting the activity of photosystem II, and inducing oxidative stress in algal cells [18,19]. As a rootless submerged macrophyte, *C. demersum* also exhibits significant algal suppression capacity. Studies have shown that *C. demersum* can effectively inhibit the growth of *Microcystis* by continuously releasing phenolic acids and fatty acids [8,20]. However, currently, few studies have comprehensively explored the remediation effects of submerged macrophytes on *Microcystis*-contaminated water from the perspective of their “competition-allelopathy-microorganisms” interactions. This study will analyze the purification effects from two aspects: “algal inhibition” and “toxin removal”, also with a particular focus on investigating the role of plant-attached microorganisms in the in situ degradation of toxins. The present study will help clarify the relative contributions of direct plant absorption, and microbial degradation to the MC removal process. By comparing the differences in inhibitory effects of the two submerged macrophytes on toxic and non-toxic algal strains, as well as the physiological changes in submerged macrophytes under the stress of toxic algae (e.g., antioxidant enzyme activities, photosynthetic characteristics), the present study also could reveal the roles and limitations of allelopathy in responding to different algal strains (especially the defensive trait of toxin production).

## 2. Materials and Methods

### 2.1. Experimental Materials

The toxic *M. aeruginosa* strains (FACHB-905) and non-toxic *M. aeruginosa* strains (FACHB-1005) used in the experiment were purchased from the Freshwater Algae Culture Collection of the Institute of Hydrobiology, Chinese Academy of Sciences (FACHB), and were cultured in sterile BG-11 medium in a constant-temperature light incubator under a 12 h:12 h light cycle (25 μmol photons s^−1^ m^−2^) for expansion until the cyanobacterial cells reached the exponential growth phase, after which they were used for the further experimentation. *C. demersum* used in the experiment was purchased from Zhengzhou Kaiximu E-Commerce Co., Ltd., Zhengzhou, China, and *M. spicatum* was purchased from Shuyang Xinweiheng Garden Co., Ltd., Suqian, China. Both plants were cleaned with a soft brush and then acclimated in hydroponic culture for one week before being used in the formal experiment. The plastic grass used in the control was purchased from Yiwu Timmei Arts and Crafts Factory, Yiwu, China. It was soaked in alcohol for 48 h, then soaked in sterile water and rinsed repeatedly before being used in the experiment.

### 2.2. Experimental Design

**Figure 1:**
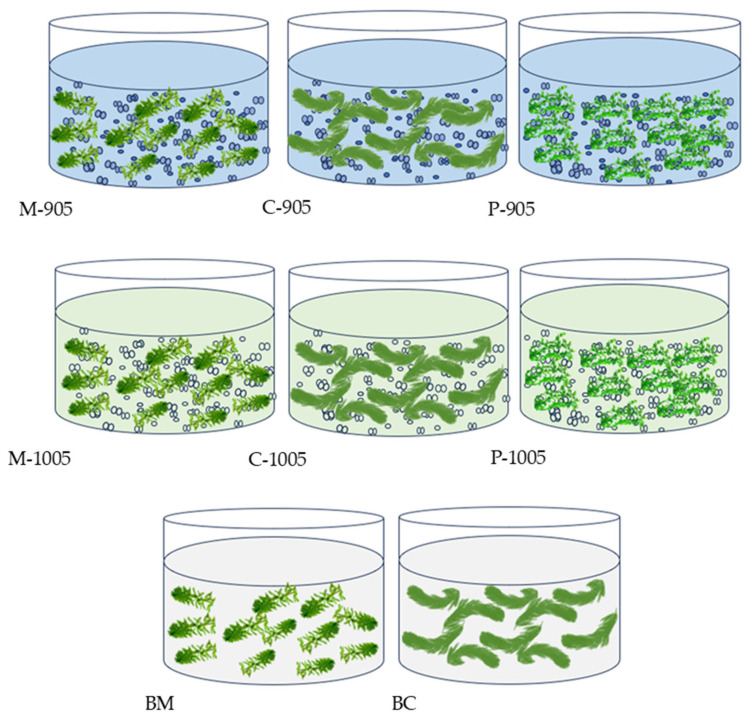
Experimental design of co-cultivation of submerged macrophytes with toxic or non-toxic *M. aeruginosa.* M-905: *M. spicatum +* Toxic *M. aeruginosa*; C-905: *C. demersum +* Toxic *M. aeruginosa*; P-905: *Plastic grass +* Toxic *M. aeruginosa*; M-1005: *M. spicatum +* Non-toxic *M. aeruginosa*; C-1005: *C. demersum +* Non-toxic *M. aeruginosa*; P-1005: *Plastic grass +* Non-toxic *M. aeruginosa*; BM: 1/10 BG-11 + *M. spicatum* (Control); BC: 1/10 BG-11 medium + *C. demersum* (Control).

A total of 4 experimental groups and 2 control groups were setup in the present study (Figure 1). The two plant species were co-cultured with water containing toxic and non-toxic *Microcystis*, respectively, as the experimental groups, while plastic grass was co-cultured with *Microcystis*-containing water as the control groups. Each of the experimental groups and control groups had 3 replicates. The experiment was conducted in 7-L cylindrical glass tanks. For each tank in the treatment groups, 5 L of *Microcystis* solution was added, and the initial optical density (OD_665_, Optical density at 665 nm) of the algal solution was adjusted to 0.1. The control groups were added with 5 L of 1/10 BG_11_ medium [21]. The initial biomass of each plant was 5 g/L, and the whole experiments lasting for 15 days under a 12 h:12 h light cycle (25 μmol photons s^−1^ m^−2^).

### 2.3. Samples Collection and Determination

Water samples were collected on days 0, 2, 4, 6, 9, 12, and 15 for the determination of total dissolved nitrogen (TDN), total dissolved phosphorus (TDP), optical density at 665 nm (OD_665_), algal density, and chlorophyll-a (Chl-a) concentration. Then, 50 mL samples were filtered through a 0.45 μm mixed-fiber aqueous filter membrane and then the filtrate were collected for determination of TDN, resorting to the potassium persulfate oxidation UV spectrophotometry method; and TDP resorting to the molybdenum-antimony anti-spectrophotometry method [22,23].

The measurement of photosynthetic pigments chlorophyll a (Chla) was carried out according to [24]. Algal density was determined at 400× magnification under an inverted microscope. The determination of catalase (CAT) activity, glutathione peroxidase (GSH-Px) activity, superoxide dismutase (SOD) activity, and malondialdehyde (MDA) content in plant tissues were determined using commercial assay kits, which were purchased from Nanjing Jiancheng Bioengineering Institute [25].

At the beginning and end of the experiment, 1 mL algal samples were collected from each group and centrifuged at 8000 r min^−1^ for 15 min. Subsequently, intracellular and extracellular microcystin was quantified using a microcystin ELISA kit purchased from the Institute of Hydrobiology, Chinese Academy of Sciences, Wuhan, China.

In addition, 1 g of plant tissue was weighed at the end of the experiment, and the surface water was gently removed with absorbent paper. The samples were placed in sterile centrifuge tubes, 10 mL 0.1 mol L^−1^ phosphate-buffered solution (PBS) was added, and ultrasonic washing was performed for 1 min, repeated for 3 times. The washing solution was collected after being washed three times and filtered with a 0.22 μm acetate fiber filter membrane. The filter membrane was placed in a 10 mL sterile centrifuge tube and stored at −80 °C for high-throughput sequencing to analyze the microbial community composition. The high-throughput sequencing service was provided by Shanghai Meiji Biomedical Technology Co., Ltd., Shanghai, China, and the community composition analysis was completed online (http://www.majorbio.com) accessed on 21 June 2025 [25,26,27,28].

### 2.4. Data Analysis

Microsoft Excel 2019 and SPSS 26.0 were used for statistical analysis of all data, while Origin Pro 2024 and GraphPad 9.2.0 were employed for data analysis and graphing. One-way ANOVA and t-tests were employed to assess the significance of differences in the removal of nutrients, Chla and microcystins by plants in each group and the antioxidative response of the plants exposure to toxic or non-toxic *M. aeruginosa*. A value of *p* < 0.05 was considered statistically significant in all analyses.

## 3. Results

### 3.1. Removal of TDN and TDP by Submerged Plants

**Figure 2:**
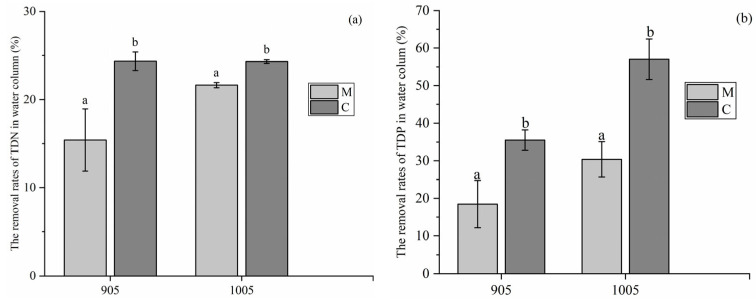
Removal rate of TDN (**a**) and TDP (**b**) by *C. demersum*, *M. spicatum* in the co-cultivation with toxic or non-toxic *M. aeruginosa* (M: treatment by *M. spicatum*; C: treatment by *C. demersum*; 905: toxic *M. aeruginosa*; 1005: non-toxic *M. aeruginosa*). Notes: the different letters in each group indicate that the differences are significant (*p* < 0.05).

As shown in Figure 2a, at the end of the experiment, *C. demersum* in the treatment groups exhibited a higher removal efficiency of TDN than *M. spicatum* in both exposure conditions to toxic and non-toxic *M. aeruginosa*. Specifically, in the cultivation with toxic *M. aeruginosa*, the TDN removal rate by *C. demersum* was 24.35%, while that by *M. spicatum* was 15.40%; in the cultivation with non-toxic *M. aeruginosa*, the TDN removal rate by *C. demersum* was 24.31%, compared with 21.62% by *M. spicatum*. Meanwhile, *C. demersum* also showed a higher TDP removal efficiency than *M. spicatum*. In detail, in the cultivation with toxic *M. aeruginosa*, the TDP removal rate by *C. demersum* reached 35.52%, whereas that by *M. spicatum* was 18.44%; in water with non-toxic *M. aeruginosa*, the DTP removal rate by *C. demersum* was 57.00%, while that by *M. spicatum* was 30.36% (Figure 2b).

### 3.2. Removal of Chlorophyll a (Chl a) by Submerged Plants

**Figure 3:**
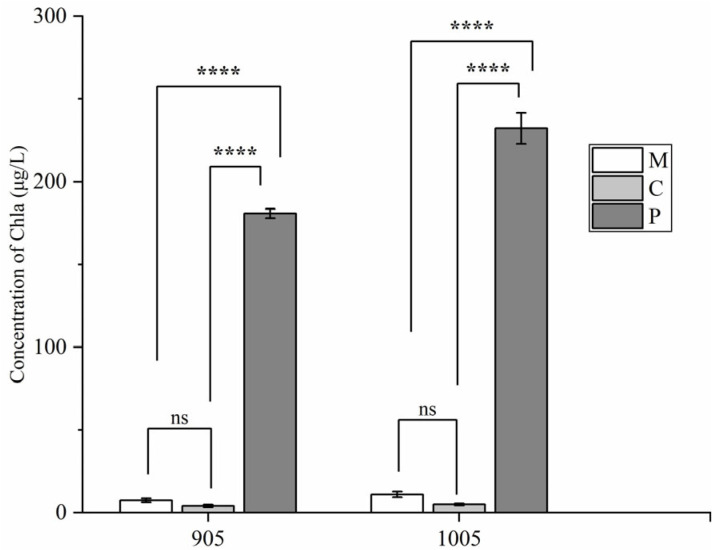
Removal of Chla by *C. demersum*, *M. spicatum* in the co-cultivation with toxic or non-toxic *M. aeruginosa* (M: treatment by *M. spicatum*; C: treatment by *C. demersum*; P: control with plastic plant). Notes: ns in each group indicate the differences are not significant (*p* > 0.05), and **** means significant differences (*p* < 0.0001).

As shown in Figure 3, at the end of the experiment, the concentration of Chla in cultivation with toxic *M. aeruginosa* treated by both plants *C. demersum* and *M. spicatum* were significantly reduced, to 7.41 μg/L and 4.08 μg/L, respectively, compared with control with plastic plants. However, no significant statistical differences were detected between the effects by two plants *C. demersum* and *M. spicatum* (*p* > 0.05). Similarly, the chlorophyll a content in the cultivation with non-toxic *M. aeruginosa* was also significantly lower than that in the control group (*p* < 0.01), decreasing to 11.05 μg/L and 4.95 μg/L, respectively. No significant statistical differences were observed between the two plants (*p* > 0.05).

### 3.3. Removal and Absorption of MC-LR (Microcystins-LR) by Submerged Plants

**Figure 4:**
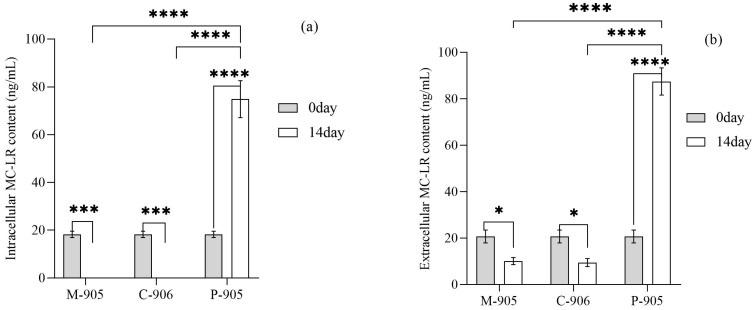
Removal of Intracellular (**a**) and Extracellular (**b**) MC-LR by submerged plants (M-905: *M. spicatum +* toxic *M. aeruginosa*; C-905: *C. demersum* + toxic *M. aeruginosa*). Notes: * in each group indicate the differences are significant (*p* < 0.05), *** means significant differences (*p* < 0.001), **** means significant differences (*p* < 0.0001).

As shown in Figure 4a, at the end of the experiment, the intracellular MC-LR content in the control group was significantly higher than that of the initial state. Compared with the control group, the intracellular MC-LR content treated by *M. spicatum* (M-905) or *C. demersum* (C-905) was significantly reduced, and at the end of the experiment, the intracellular MC-LR content in both plants treatment groups reached to 0 ng/mL. Similarly, the extracellular MC-LR content in the control group (P-905) was significantly higher than that that of the initial state, reaching 87.44 ng/mL. In contrast, the extracellular MC-LR content in the M-905 and C-905 treatment at the end of the experiment was significantly lower than that of the control group with plastic plants, decreasing to 10.09 ng/mL and 9.47 ng/mL, respectively (Figure 4b).

**Figure 5:**
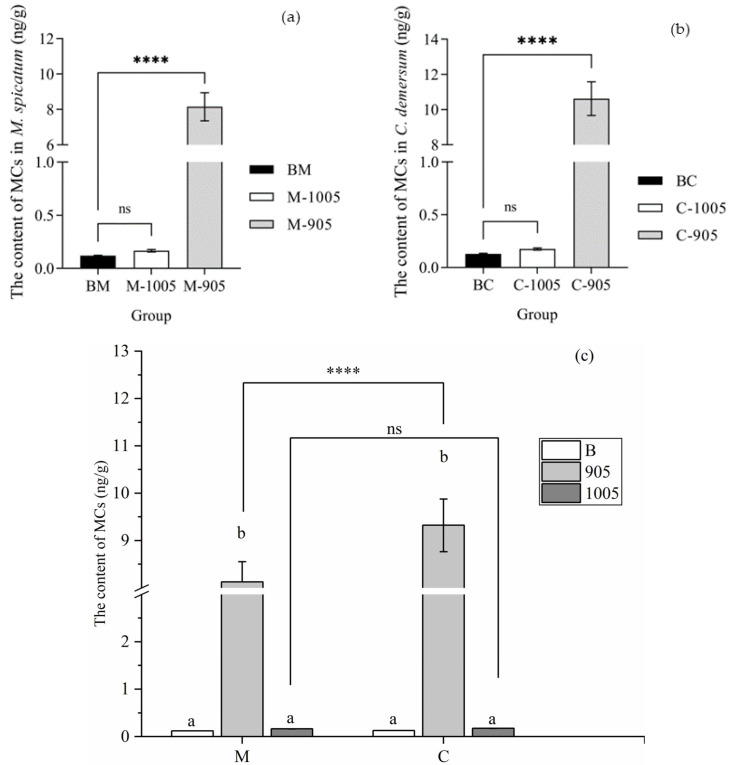
The MC-LR content in the tissues of *M. spicatum* (**a**), *C. demersum* (**b**), and comparison of *M. spicatum*, *C. demersum* (**c**). (BM: BG-11 *+ M. spicatum*; M-905: *M. spicatum +* toxic *M. aeruginosa*; M-1005: *M. spicatum +* non-toxic *M. aeruginosa*; BC: BG-11 *+ C. demersum*; C-905: *C. demersum +* toxic *M. aeruginosa*; C-1005: *C. demersum +* non-toxic *M. aeruginosa*). Notes: ns in each group indicate the differences are not significant (*p* > 0.05), **** means significant differences (*p* < 0.0001), and the same letters in each group indicate that the differences are not significant (*p* > 0.05), different letters indicate significant differences (*p* < 0.05).

The MC-LR accumulation capacity of *C. demersum* was significantly higher than that of *M. spicatum*. As shown in Figure 5, after 14 days of exposure to toxic *M. aeruginosa*, the MCs content in *M. spicatum* tissues was 8.13 ng/g fresh weight (FW), while that in *C. demersum* tissues was 9.32 ng/g FW.

### 3.4. Antioxidant Response of Submerged Macrophytes to Toxic or Non-Toxic M. aeruginosa

**Figure 6:**
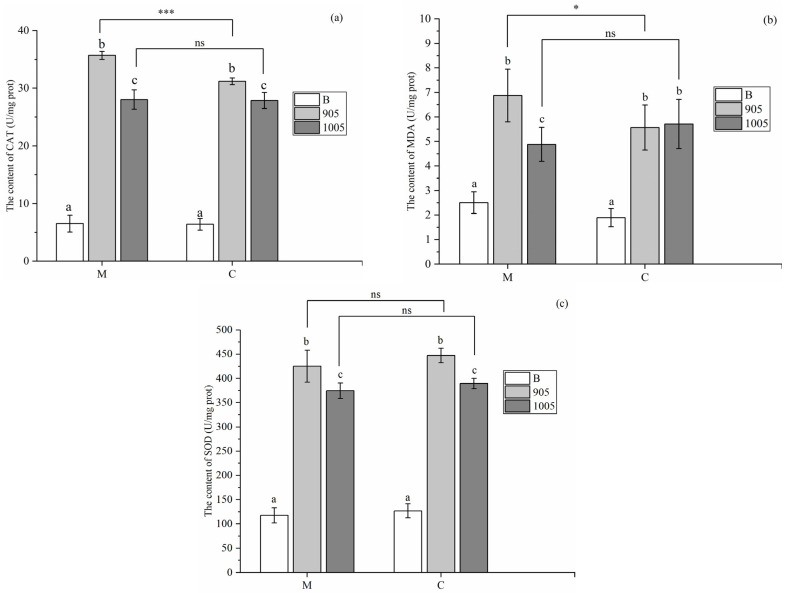
Content of CAT (**a**), MDA (**b**), and SOD (**c**) in *M. spicatum* and *C. demersum* under exposure to toxic or non-toxic *M. aeruginosa* (B: BG-11 *medium*; 905: toxic *M. aeruginosa*; 1005: non-toxic *M. aeruginosa*; M: *M. spicatum*; C: *C. demersum*). Notes: ns in each group indicate the differences are not significant (*p* > 0.05), * means significant differences (*p* < 0.05), *** means significant differences (*p* < 0.001) and the same letters in each group indicate that the differences are not significant (*p* > 0.05), different letters indicate significant differences (*p* < 0.05).

At the end of the experiment, the catalase (CAT) activity of *M. spicatum* was 36.17 U/mg prot and 26.11 U/mg prot exposure to toxic *M. aeruginosa* or non-toxic *M. aeruginosa*, respectively, compared with 6.52 U/mg prot in the control group. For *C. demersum*, the CAT activity was 31.86 U/mg prot and 28.43 U/mg prot exposure to toxic *M. aeruginosa* or non-toxic *M. aeruginosa*, respectively, compared with 7.51 U/mg prot in the control group. Statistical analysis showed that the CAT activity of *M. spicatum* was significantly higher than that of *C. demersum* under toxic *Microcystis* exposure, while no significant difference was observed under non-toxic *Microcystis* exposure (*p* > 0.05) (Figure 6a).

Statistical analysis also revealed that the MDA content in *M. spicatum* was significantly higher than that of *C. demersum* under toxic *M. aeruginosa* exposure, whereas no significant difference existed between the two plants exposure to non-toxic *M. aeruginosa* exposure (*p* > 0.05). As it was shown in Figure 6b, the MDA content of *M. spicatum* was 4.88 U/mg prot exposure to non-toxic *M. aeruginosa* and 7.87 U/mg prot exposure to toxic *M. aeruginosa*, compared that with 2.5 U/mg prot in the control group. The MDA content of *C. demersum* was 5.71 U/mg prot exposure to non-toxic *M. aeruginosa* and 5.57 U/mg prot exposure to toxic *M. aeruginosa*, compared with 1.89 U/mg prot in the control group.

At the end of the experiment, the superoxide dismutase (SOD) activity of *M. spicatum* treated with *Microcystis* was significantly elevated. Specifically, the SOD activity of *M. spicatum* was 117.57 U/mg prot in the control group without *Microcystis* exposure, while it reached 425.06 U/mg prot, 374.36 U/mg prot, respectively, exposure to toxic or non-toxic *M. aeruginosa*, respectively. Meanwhile, the SOD activity of *C. demersum* treated with *Microcystis* was also significantly elevated, compared with no *Micsocystis* exposure. The SOD activity of *C. demersum* was 389.45 U/mg prot exposure to non-toxic *M. aeruginosa* and 447.1 U/mg prot exposure to toxic *M. aeruginosa*, compared with 126.91 U/mg prot in the control group. Statistical analysis also revealed that both *M. spicatum* and *C. demersum* exhibited significant differences between the SOD activity exposure to toxic and non-toxic *M. aeruginosa* (Figure 6c).

### 3.5. The Response of Microorganisms to Toxic and Non-Toxic M. aeruginosa

**Figure 7:**
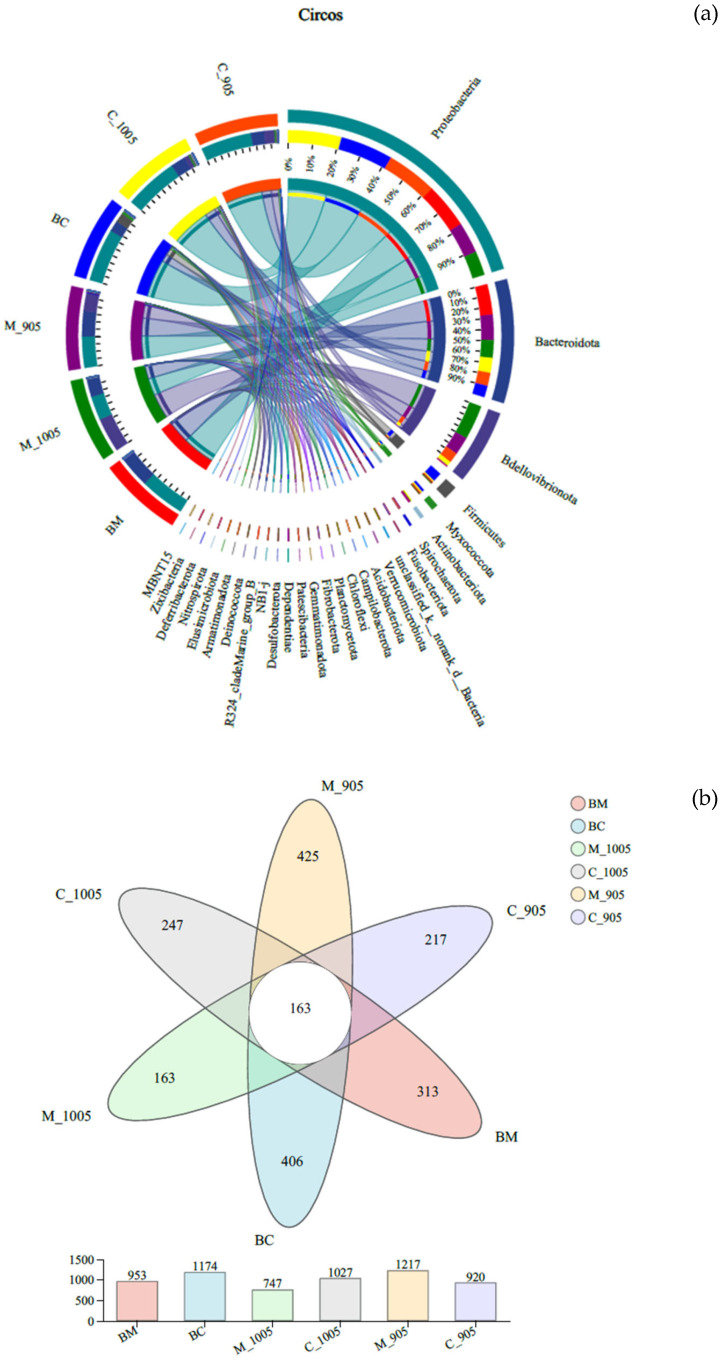
Circos plot (**a**) and Venn diagram (**b**) of bacterial communities. (BM: BG-11 medium + *M. spicatum*; M_1005: Non-toxic *M. aeruginosa* + *M. spicatum*; M_905: Toxic *M. aeruginosa* + *M. spicatum*; BC: BG-11 medium + *C. demersum*; C_1005: Non-toxic *M. aeruginosa* + *C. demersum*; C_905: Toxic *M. aeruginosa* + *C. demersum*).

The Circos plot illustrates the distribution of microbial taxa across different microbial samples (Figure 7a). Among the six groups, Proteobacteria and Bacteroidota were the dominant phyla, accounting for 9–21% and 10–27%, respectively. Specifically, Bdellovibrionota had a relatively high proportion in the non-toxic *M. aeruginosa* + *M. spicatum* group and the toxic *M. aeruginosa* + *M. spicatum* group, representing 51% and 27% of the community, respectively. Firmicutes exhibited the highest proportion in the BG-11 + *C. demersum* group, up to 75%. Meanwhile, Myxococcota was relatively abundant in the BG-11 + *C. demersum* (control) group, non-toxic *M. aeruginosa* + *C. demersum* group, and toxic *M. aeruginosa* + *C. demersum* group, accounting for 47%, 26%, and 23%, respectively. As shown in the Venn diagram (Figure 7b), the six treatment groups shared 163 operational taxonomic units (OTUs). Among them, the toxic *M. aeruginosa* + *M. spicatum* group had the highest number of unique OTUs (425), with a total of 1217 OTUs. Followed by the BG-11 + *C. demersum* group, which had 406 unique OTUs and a total of 1174 OTUs. The non-toxic *M. aeruginosa* + *M. spicatum* group had 163 unique OTUs and a total of 747 OTUs, which was the lowest total number of OTUs among all groups.

**Figure 8:**
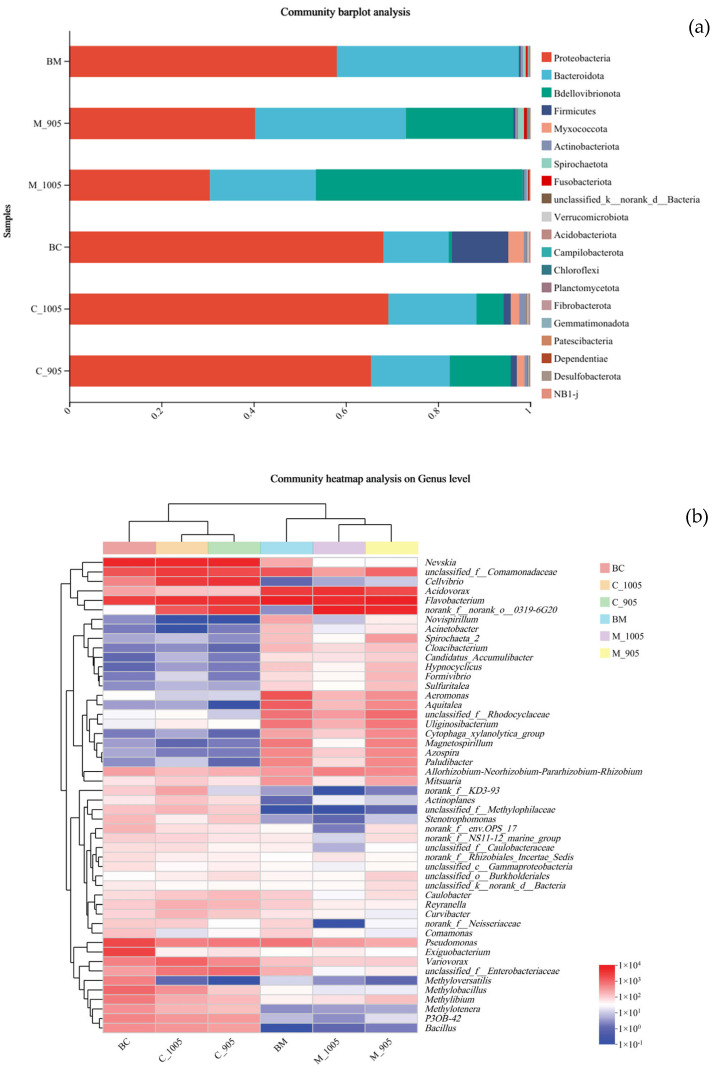
Communities composition at the phylum level (**a**) and the heatmap at the genus level (**b**) for different experimental groups. BM: BG-11 medium + *M. spicatum* (control); M_1005: Non-toxic *M. aeruginosa* + *M. spicatum*; M_905: Toxic *M. aeruginosa* + *M. spicatum*; BC: BG-11 medium + *C. demersum* (control); C_1005: Non-toxic *M. aeruginosa* + *C. demersum*; C_905: Toxic *M. aeruginosa* + *C. demersum*.

As shown in Figure 8a, the dominant phyla of the microorganisms across the 6 groups were Proteobacteria (30.5–69.3%) and Bacteroidota (14.1–39.5%). Specifically, Bdellovibrionota had a relatively high proportion in the non-toxic *M. aeruginosa* + *M. spicatum* group and the toxic *M. aeruginosa* + *M. spicatum* group, accounting for 44.8% and 23.4%, respectively, which was significantly higher than that in the BG-11 + *M. spicatum* group. Notably, Firmicutes exhibited the highest proportion (12.2%) in the BG-11 + *C. demersum* group, which was significantly higher than those in the non-toxic *M. aeruginosa* + *C. demersum* group and toxic *M. aeruginosa* + *C. demersum* group. The heatmap in Figure 8b illustrated the genus-level differences in microorganisms between each treatment and the control group. A total of the top 50 microbial genera were identified. Among these, *Flavobacterium*, *Acidovorax*, and *Pseudomonas* were the absolute dominant genera in all treatment groups, with their abundances significantly higher than those of other genera. The microbial abundance varied across different treatments. It was indicated that the abundances of *Cellvibrio*, *Methylotenera* and *Bacillus* attached to *C. demersum* were significantly higher than those attached to *M. spicatum*. In contrast, the abundances of *Aeromonas*, *Aquitalea*, *unclassified_f__Rhodocyclaceae*, *Azospira*, and *Paludibacter* attached to *M. spicatum* were significantly higher than those attached to *C. demersum*. These results indicated that there were significant differences in the composition of microorganisms attached to different plant species.

**Figure 9:**
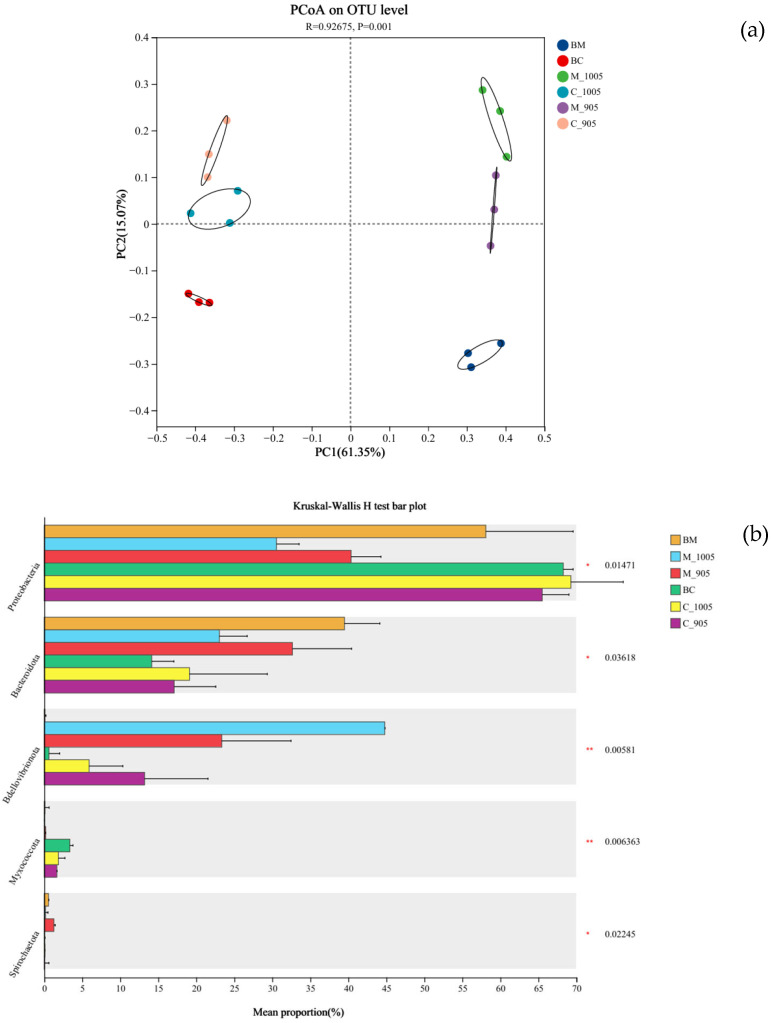
PCoA analysis at the OTU level (**a**) and Bar Chart of inter-group differences at the genus level (**b**) for different groups (BM: BG-11 + *M. spicatum*; M_1005: Non-toxic *M. aeruginosa* + *M. spicatum*; M_905: Toxic *M. aeruginosa* + *M. spicatum*; BC: BG-11 + *C. demersum*; C_1005: Non-toxic *M. aeruginosa* + *C. demersum*; C_905: Toxic *M. aeruginosa* + *C. demersum;* * *p* < 0.05, ** *p* < 0.01).

The PCoA plot was used for comparative analysis of OTUs among groups to assess the diversity of microbial communities at the phylum level (Figure 9a), aiming to explore differences in bacterial community composition between each treatment group. The results of the PCoA analysis (PERMANOVA, R^2^ = 0.9267, *p* = 0.001) revealed a clear separation in bacterial communities between the groups BG-11 + *M. spicatum* and non-toxic *M. aeruginosa* + *M. spicatum* and toxic *M. aeruginosa* + *M. spicatum*; between BG-11 + *C. demersum* and non-toxic *M. aeruginosa* + *C. demersum* and toxic *M. aeruginosa* + *C. demersum*. The results indicated that *M. aeruginosa* had a significant effect on the composition of microbial communities attached to the submerged macrophytes. Furthermore, based on the inter-group difference test analysis of bacterial genera associated with different plants (Tukey–Kramer test), it was also indicated that *Proteobacteria*, *Bacteroidota*, *Bdellovibrionota*, *Myxococcota*, and *Spirochaetota* exhibited extremely significant differences among each group (Tukey–Kramer, * *p* < 0.05, ** *p* < 0.01, *** *p* < 0.001) (Figure 9b).

## 4. Discussion

### 4.1. Removal Efficiency of Nutrients and Chl a Content by Submerged Plants

Chlorophyll a, a key photosynthetic pigment indicating algal biomass, is widely used for monitoring and assessing eutrophication in water bodies. The content changes are directly related to the water quality and ecological balance of aquatic ecosystems. Previous studies have shown that submerged macrophytes can inhibit the growth of *Microcystis* through multiple pathways: on the one hand, the plants form a covering layer on the water surface, creating a light-shading effect that reduces light intensity in the water, thereby inhibiting the photosynthesis of *Microcystis* and restricting its growth. On the other hand, submerged macrophytes can secrete allelochemicals, which directly inhibit the growth rate of algae and interfere with the synthesis of their photosynthetic pigments [29]. In this study, it was also found that *C. demersum* and *M. spicatum* could significantly reduce the concentration of Chla in *Microcystis*-containing water, but there was no significant difference in this allelopathy effect between toxic and non-toxic *Microcystis*. Intense nutrient competition is likely the core reason for this phenomenon. Both *C. demersum* and *M. spicatum* have been confirmed as species with high efficiency in absorbing nitrogen and phosphorus from water; they probably deplete available nutrients in the water rapidly, thereby imposing equal and fundamental restrictions on the growth of all types of *Microcystis* [10]. Furthermore, whether *Microcystis* produces toxins or not had no differential impacts on the inhibition of algae by submerged macrophytes, which may also be attributed to the role of algal toxins in plant-algal competition, the importance of which was often secondary to resource competition. Therefore, in the present study, regardless of whether *Microcystis* produced toxins or not, their growth were successfully inhibited by the two plant species with equal effectiveness.

The results of this study also indicated that both *M. spicatum* and *C. demersum* exhibit significant effectiveness in removing soluble nitrogen and phosphorus from *Microcystis*-containing water [14,30]. However, there was a notable difference in their nitrogen and phosphorus removal capacities. *C. demersum* showed superior performance in removing soluble nitrogen and phosphorus compared to *M. spicatum,* which may be related to the morphological characteristics of the two plants species. As we all know, *C. demersum* lacks roots, and its leaves are highly dissected throughout the plant. This morphology gives it one of the largest specific surface areas among all submerged macrophytes, providing a substantial contact area for nutrient absorption. In contrast, although *M. spicatum* also has dissected leaves, its leaf structure may be more fragile and prone to damage under stressful conditions, which impairs its functional performance. In addition, it was also found in the present study that both *C. demersum* and *M. spicatum* achieved higher nitrogen and phosphorus removal rates in water with non-toxic *Microcystis* than in water with toxic *Microcystis*. This result can be explained by the physiological stress exerted on submerged macrophytes by microcystins released by toxic *Microcystis*. Multiple studies have confirmed that microcystins (e.g., MC-LR) can induce oxidative stress in submerged macrophytes, damage their chloroplast structure and cell membrane integrity, and significantly inhibit their relative growth rate [17,25,31]. In water containing toxic *Microcystis*, plants must consume substantial energy to resist toxin-induced stress (e.g., activating antioxidant systems, repairing cell damage), which inevitably would reduce the energy available for growth and nutrient absorption, leading to decreased nitrogen and phosphorus removal efficiency. Based on this, we hypothesized that in water dominated by toxic *Microcystis*, submerged macrophytes must allocate more energy to coping with toxin stress and maintaining survival, rather than to growth and nutrient absorption. This ultimately resulted in diminished water purification capacity for soluble nitrogen and phosphorus.

### 4.2. The Removal and Absorption of MC-LR by Submerged Macrophytes

In this study, it was found that in toxic *Microcystis*-containing water treated with *C. demersum* and *M. spicatum*, almost all algal cells were lysed by the end of the experiment, and the intracellular toxin concentration dropped to an extremely low level. Meanwhile, the concentration of extracellular toxins in the water was also significantly lower than that in the plant-free control group, and bioaccumulation of microcystins was detected in the tissues of both plant species [32]. also indicated that the submerged macrophytes including *Egeria densa*, *C. demersum*, and *M. aquaticum* could achieve a 100% removal rate of MC-LR from the reservoir water (a drinking water source) within two weeks. It was suggested that the submerged plants might accomplish the reduction in MC-LR through pathways such as absorption or promotion of microbial degradation [25]. reported that *Vallisneria natans* could also rapidly adsorb and absorb MC-LR from water; meanwhile, the structure of the microbial community attached to its roots changed, exhibiting a stronger potential for MC-LR degradation. Based on this, *C. demersum* and *M. spicatum* might remove toxins released by lysed algal cells through similar pathways—i.e., the combined effect of absorption by the plants themselves and degradation by attached microorganisms [21,33]. Furthermore, submerged macrophytes might serve as an important “sink” for microcystins in aquatic ecosystems. They transfer and immobilize toxins from water into their tissues via active or passive absorption. In this study, the microcystin bioaccumulation capacity of both *C. demersum* and *M. spicatum* was also detected; notably, *C. demersum* exhibited a higher bioaccumulation capacity than *M. spicatum*, which may be attributed to its large specific surface area (rootless and highly dissected leaves).

### 4.3. Antioxidant Response of the Submerged Macrophytes to Toxic and Non-Toxic M. aeruginosa

Submerged macrophytes exhibited significant changes in antioxidant responses during the whole experimentation. Catalase (CAT), a key enzyme in reactive oxygen species (ROS) metabolism [34], could efficiently break down hydrogen peroxide (H_2_O_2_), which is toxic to plants, into harmless water and oxygen. H_2_O_2_ is not only a metabolic by-product but also an important signaling molecule involved in regulating plant growth and development as well as responding to environmental stress. Malondialdehyde (MDA), the end product of lipid peroxidation, whose changes can directly reflect the degree of oxidative damage to proteins and cell membrane systems in plants under abiotic and biotic stress, serves as a crucial indicator for evaluating the physiological state of plants under stress [35]. Superoxide dismutase (SOD), a core component of the enzymatic antioxidant system, plays a key role in the primary defense of plants against environmental stress by catalyzing the dismutation of superoxide anion radicals [36]. The results of this study showed that the activities of CAT and SOD, as well as the content of MDA, in both *C. demersum* and *M. spicatum* were significantly increased both in the presence of toxic and non-toxic *M. aeruginosa*. This indicated that the stress by *Microcystis* could induce membrane lipid peroxidation, and the plants responded by activating their antioxidant enzyme systems. The antioxidant response under non-toxic *Microcystis* also suggested that the presence of *Microcystis* itself could impose stress on submerged macrophytes (e.g., nutrient competition, physical shading), thereby inducing an active antioxidant defense response in the plants. In this study, we observed that under toxic *M. aeruginosa* stress, the contents of CAT and MDA in both plant species were significantly higher than those in the non-toxic group. This directly confirmed that microcystins are key factors exacerbating oxidative damage in plants, which was consistent with the findings of [37,38]. These studies similarly found that the levels of SOD, CAT, peroxidase (POD), and glutathione (GSH) in *V. natans* were significantly induced and increased by MC-LR. Additionally, we found that the contents of CAT and MDA in *M. spicatum* were higher than those in *C. demersum* across all treatment groups. This suggested that under the same toxin stress, *M. spicatum* might suffer more severe oxidative damage, or its stress response is more intense, further implying that *M. spicatum* might have lower tolerance than that of *C. demersum*.

### 4.4. Response of Attached Microorganisms of the Plants to Microcystis

By analyzing the epiphytic microbial community structure of submerged macrophytes, this study revealed that plant species and algal stress (especially whether *Microcystis* is toxic or not) are likely the two core factors driving microbial community assembly. These specific microbial communities may be directly involved in the physiological processes of plant-mediated algal inhibition and detoxification. The present study systematically analyzed the epiphytic microbial community structure of submerged macrophytes across different treatment groups using high-throughput sequencing technology. A total of 28 phyla, 59 classes, 144 orders, 251 families, 451 genera, and 710 species of microorganisms were detected. Among these, Proteobacteria and Bacteroidota were the dominant phyla in all groups, which was consistent with previous findings on microbial composition in aquatic ecosystems. These two phyla are known for their metabolic diversity and participate in the cycling of multiple elements such as carbon, nitrogen, and sulfur, serving as core microbial groups that maintain the basic functions of the ecosystem [39]. Proteobacteria are capable of utilizing various carbon sources and thus can grow in different environments. By exerting their biochemical properties, they contribute to plant growth, enhance plant tolerance to heavy metals, and improve plant remediation capabilities [40]. Bacteroidota are abundant pathogen-inhibiting members of the plant microbiome. They can inhibit the growth of pathogenic bacteria in water, enhance plant disease resistance, and act as key regulators of microbiome function. Additionally, they can degrade sugars in plants and produce organic acids [41].

Notably, regarding the number of unique OTUs across treatment groups, the toxic *M. aeruginosa* + *M. spicatum* group had the highest number of unique OTUs (425) and total OTUs. This strongly suggested that this treatment group formed the most specific and complex microbial ecosystem. This may be because, under toxic algal stress, *M. spicatum* secreted specific compounds (e.g., stress metabolites or specific allelochemicals) through its roots, recruiting a unique, functionally specialized microbial community to assist in resisting stress. In contrast, the non-toxic *M. aeruginosa* + *M. spicatum* group had the lowest number of unique OTUs and total OTUs, indicating that the microbial environment was relatively simple in the absence of toxin stress. A more important finding was the plant-specificity observed at the genus level: *Flavobacterium* (often capable of degrading complex organic matter) and *Pseudomonas* (known for its diverse metabolic capabilities, including degrading pollutants, toxins, and producing antibiotics) [42,43] were absolutely dominant in all groups. This indicated that they are highly conserved and functionally important core microbial taxa symbiotic with submerged macrophytes. Additionally, heatmap analysis clearly revealed that different plants possess unique “microbial fingerprints.” For example, *Cellvibrio* (capable of degrading cellulose and chitin) and *Bacillus* (a typical representative of *Firmicutes*, which can produce various antimicrobial substances and degrade toxins) [42]. were significantly enriched in the *C. demersum* groups. In contrast, *Aquitalea* and *Azospira* (associated with nitrogen fixation) were more abundant in the *M. spicatum* groups. This difference strongly suggested that *C. demersum* and *M. spicatum* recruited functionally distinct, specific microbial communities to assist in adapting to the environment. A key finding was that Bdellovibrionota was abnormally enriched (up to 44.8%) in the *M. spicatum* groups, particularly in the non-toxic *Microcystis* group. Bacteria of this phylum are obligate predatory/parasitic bacteria that can attack, invade, and lyse various Gram-negative bacteria, including cyanobacteria. This result provided direct evidence of *M. spicatum*—mediated algal inhibition from a microbiological perspective: *M. spicatum* may be particularly adept at creating a rhizosphere microenvironment conducive to the growth of such natural “algal killers,” indirectly inhibiting *Microcystis* growth by activating the “microbial predation” pathway. In contrast, the relatively high abundance of *Firmicutes* in the *C. demersum* control group, as well as the enrichment of genera such as *Bacillus*, suggested that it may rely more on pathways such as allelopathic inhibition or nutrient competition. This inter-specific differentiation in microbial functions provided a new perspective for explaining the differences between the two plant species in terms of algal inhibition efficiency and physiological stress responses.

The results of the PCoA analysis (PERMANOVA, R^2^ = 0.9267, *p* = 0.001) provided compelling evidence that significant separation in epiphytic microbial community structure occurred between the control groups and each *Microcystis*-treated group of both *M. spicatum* and *C. demersum*. This confirmed that the introduction of *Microcystis* has strongly reshaped the epiphytic microbial communities of the plants. This reshaping can be interpreted as a shift in the plant microbiome from a “steady-state” to a “stress-induced” state. Under algal stress, plants may actively recruit beneficial microorganisms that assisted in stress resistance (e.g., aiding in microcystin degradation, resisting oxidative stress, or participating in nutrient competition) by altering the composition of their root exudates. Statistically highly significant differentials in Bdellovibrionota and Myxococcota further confirmed that these taxa serve as key microbial indicators in response to algal stress.

## Data Availability

The original contributions presented in this study are included in this article. Further inquiries can be directed to the corresponding authors.

## References

[1] Paerl H.W., Paul V.J. (2012). Climate change: Links to global expansion of harmful cyanobacteria. Water Res..

[2] De Figueiredo D.R., Azeiteiro U.M., Esteves S.M., Gonalves F.J., Pereira M.J. (2004). Microcystin-producing blooms-a serious global public health issue. Ecotoxicol. Environ. Saf..

[3] Omidi A., Esterhuizen-Londt M., Pflugmacher S. (2018). Still challenging: The ecological function of the cyanobacterial toxin microcystin—What we know so far. Toxin Rev..

[4] Pham T.L., Utsumi M. (2018). An overview of the accumulation of microcystins in aquatic ecosystems. J. Environ. Manag..

[5] Park J., Church J., Son Y., Kim K.T., Lee W.H. (2017). Recent advances in ultrasonic treatment: Challenges and field applications for controlling harmful algal blooms (HABs). Ultrason. Sonochemistry.

[6] Jančula D., Maršálek B. (2011). Critical review of actually available chemical compounds for prevention and management of cyanobacterial blooms. Chemosphere.

[7] Bittencourt-Oliveira M.D.C., Chia M.A., Oliveira H.S.B.D., Araújo M.K.C., Molica R.J.R., Dias C.T.S. (2015). Allelopathic interactions between microcystin-producing and non-microcystin-producing cyanobacteria and green microalgae: Implications for microcystins production. J. Appl. Phycol..

[8] Amorim C.A., Moura A.N. (2020). Effects of the manipulation of submerged macrophytes, large zooplankton, and nutrients on a cyanobacterial bloom: A mesocosm study in a tropical shallow reservoir. Environ. Pollut..

[9] Li B., Yin Y., Kang L., Feng L., Liu Y., Du Z., Tian Y., Zhang L. (2021). A review: Application of allelochemicals in water ecological restoration—Algal inhibition. Chemosphere.

[10] Wang D., Gan X., Wang Z., Jiang S., Zheng X., Zhao M., Zhang Y., Fan C., Wu S., Du L. (2023). Research status on remediation of eutrophic water by submerged macrophytes: A review. Process Saf. Environ. Prot..

[11] Blindow I., Hargeby A., Hilt S. (2014). Facilitation of clear-water conditions in shallow lakes by macrophytes: Differences between charophyte and angiosperm dominance. Hydrobiologia.

[12] Hilt S., Gross E.M. (2008). Can allelopathically active submerged macrophytes stabilise clear-water states in shallow lakes?. Basic Appl. Ecol..

[13] Zhou Y., Zhou X., Han R., Xu X., Wang G., Liu X., Bi F., Feng D. (2017). Reproduction capacity of Potamogeton crispus fragments and its role in water purification and algae inhibition in eutrophic lakes. Sci. Total Environ..

[14] Chao C., Wang L., Li Y., Yan Z., Liu H., Yu D., Liu C. (2021). Response of sediment and water microbial communities to submerged vegetations restoration in a shallow eutrophic lake. Sci. Total Environ..

[15] Nakai S., Inoue Y., Hosomi M., Murakami A. (2000). Myriophyllum spicatum-released allelopathic polyphenols inhibiting growth of blue-green algae Microcystis aeruginosa. Water Res..

[16] Jiang M., Zhou Y., Wang N., Xu L., Zheng Z., Zhang J. (2019). Allelopathic effects of harmful algal extracts and exudates on biofilms on leaves of Vallisneria natans. Sci. Total Environ..

[17] Dong J., Dai D., Yang Y., Wang F., Zhang Y., Zhang M., Gao Y., Gao X., Li X. (2023). Growth and morphological responses of Scenedesmus obliquus to submerged macrophyte Egeria densa. Aquat. Ecol..

[18] Shao J., Wu Z., Yu G., Peng X., Li R. (2009). Allelopathic mechanism of pyrogallol to *Microcystis aeruginosa* PCC7806 (Cyanobacteria): From views of gene expression and antioxidant system. Chemosphere.

[19] Zhu J., Liu B., Wang J., Gao Y., Wu Z. (2010). Study on the mechanism of allelopathic influence on cyanobacteria and chlorophytes by submerged macrophyte (*Myriophyllum spicatum*) and its secretion. Aquat. Toxicol..

[20] Xian Q., Chen H., Liu H., Zou H., Yin D. (2006). Isolation and identification of antialgal compounds from the leaves of *Vallisneria spiralis* L. by activity-guided fractionation (5 pp). Environ. Sci. Pollut. Res..

[21] Dong J., Yang Y., Dai D., Wang F., Zhang Y., Chen Y., Yuan J., Guo C., Zhang M., Gao X. (2022). Response of submerged macrophyte *Ceratophyllum demersum* to the exponential phase (EP) and declining phase (DP) of toxic *Microcystis aeruginosa*. Hydrobiologia.

[22] Huang X.F., Chen W., Cai Q. (2000). Survey, Observation and Analysis of Lake Ecosystem.

[23] Cai Q. (2007). Protocols for Standard Observation and Measurement in Aquatic Ecosystems.

[24] Lichtenthaler H.K., Buschmann C. (2001). Extraction of phtosynthetic tissues: Chlorophylls and carotenoids. Curr. Protoc. Food Anal. Chem..

[25] Li Q., Gu P., Zhang C., Luo X., Zhang H., Zhang J., Zheng Z. (2020). Combined toxic effects of anatoxin-a and microcystin-LR on submerged macrophytes and biofilms. J. Hazard. Mater..

[26] Li Q., Gu P., Zhang H., Luo X., Zhang J., Zheng Z. (2020). Response of submerged macrophytes and leaf biofilms to the decline phase of *Microcystis aeruginosa*: Antioxidant response, ultrastructure, microbial properties, and potential mechanism. Sci. Total Environ..

[27] Sha Y., Zhang S., Dong J., Gao X., Yuan H., Zhang J., Gao Y., Li X. (2024). Effects of Toxic and Non-Toxic *Microcystis aeruginosa* on the Defense System of *Ceratophyllum demersum*–*Scenedesmus obliquus*. Microorganisms.

[28] Zhang S., Sha Y., Tang Y., Li L., Wang F., Dong J., Li X., Gao Y., Gao X., Yuan H. (2024). Laboratory-simulated inhibitory effects of the floating-bed plants on *Microcystis aeruginosa* and their microbial communities’ responses to microcystins. Microorganisms.

[29] Mohamed Z.A. (2017). Macrophytes-cyanobacteria allelopathic interactions and their implications for water resources management—A review. Limnologica.

[30] Li W., Zhang Z., Ashraf M. (2018). Allelopathic effects of various aquatic plants in eutrophic water areas. J. Coast. Res..

[31] Rojo C., Segura M., Cortés F., Rodrigo M.A. (2013). Allelopathic effects of microcystin-LR on the germination, growth and metabolism of five charophyte species and a submerged angiosperm. Aquat. Toxicol..

[32] Loise de Morais Calado S., Esterhuizen-Londt M., Cristina Silva de Assis H., Pflugmacher S. (2019). Phytoremediation: Green technology for the removal of mixed contaminants of a water supply reservoir. Int. J. Phytoremediation.

[33] Dong J., Dai D., Yang Y., Wang F., Li X., Yuan J., Chen Y., Gao Y., Zhang M., Gao X. (2022). Responses of submerged macrophyte *Ceratophyllum demersum* to the gradient concentrations of microcystin-LR (MC-LR). Environ. Sci. Pollut. Res..

[34] Nene T., Yadav M., Yadav H.S. (2022). Plant catalase in silico characterization and phylogenetic analysis with structural modeling. J. Genet. Eng. Biotechnol..

[35] Kumar A., Prasad A., Sedlářová M., Pospíšil P. (2023). Malondialdehyde enhances PsbP protein release during heat stress in Arabidopsis. Plant Physiol. Biochem..

[36] Wang Y., Branicky R., Noë A., Hekimi S. (2018). Superoxide dismutases: Dual roles in controlling ROS damage and regulating ROS signaling. J. Cell Biol..

[37] Ge J., Li J., Zhang J., Yang Z. (2012). Time-dependent oxidative stress responses of submerged macrophyte Vallisneria natans seedlings exposed to ammonia in combination with microcystin under laboratory conditions. Bull. Environ. Contam. Toxicol..

[38] Jin J., Gu X., Song R., Wang X., Yang L. (2011). Microcystin-LR induced oxidative stress and ultrastructural alterations in mesophyll cells of submerged macrophyte *Vallisneria natans* (Lour.). Hara. J. Hazard. Mater..

[39] Liu J., Li J., Wang X., Zhang Q., Littleton H. (2017). Rapid aerobic granulation in an SBR treating piggery wastewater by seeding sludge from a municipal WWTP. J. Environ. Sci..

[40] Martínez-Martínez J.G., Rosales-Loredo S., Hernández-Morales A., Arvizu-Gómez J.L., Carranza-Álvarez C., Macías-Pérez J.R., Rolón-Cárdenas G.A., Pacheco-Aguilar J.R. (2023). Bacterial communities associated with the roots of *Typha* spp. and its relationship in phytoremediation processes. Microorganisms.

[41] Lidbury I.D.E., Borsetto C., Murphy A.R.J., Bottrill A., Jones A.M.E., Bending G.D., Hammond J.P., Chen Y., Wleeington E.M.H., Scanlan D.J. (2021). Niche-adaptation in plant-associated *Bacteroidetes* favours specialisation in organic phosphorus mineralisation. ISME J..

[42] Jones G.J., Orr P.T. (1994). Release and degradation of microcystin following algicide treatment of a *Microcystis aeruginosa* bloom in a recreational lake, as determined by HPLC and protein phosphatase inhibition assay. Water Res..

[43] Jin L., Ko S.R., Ahn C.Y., Lee H.G., Oh H.M. (2016). *Rhizobacter profundi* sp. nov., isolated from freshwater sediment. Int. J. Syst. Evol. Microbiol..

